# Self-care and Health Care in Postpartum Women with Obesity: A Qualitative Study

**DOI:** 10.1055/s-0039-3400456

**Published:** 2020-01

**Authors:** Débora Bicudo Faria-Schützer, Fernanda Garanhani Surita, Larissa Rodrigues, Daiane Sofia de Morais Paulino, Egberto Ribeiro Turato

**Affiliations:** 1 Department of Tocogynecology, Universidade Estadual de Campinas, Campinas, SP, Brazil; 2 Department of Medium Psychology and Psychiatry, Universidade Estadual de Campinas, Campinas, SP, Brazil

**Keywords:** self-care, health care, postpartum, childbirth, obesity, psychologycal issues, autocuidado, cuidados de saúde, pós-parto, parto, obesidade, aspectos psicológicos

## Abstract

**Objective** To explore the experiences of women with obesity regarding self-care and the care provided by their families and health team after childbirth.

**Methods** A clinical qualitative study performed at the Postnatal Outpatient Clinic of Hospital da Mulher, Universidade Estadual de Campinas, Brazil. The sample was selected using the saturation criteria, with 16 women with obesity up to 6 months after childbirth.

**Results** The analysis comprised three categories: 1) postnatal self-care; 2) family support for woman after childbirth; and 3) postnatal health care service for women with obesity.

**Conclusion** Women with obesity need support from the health team and from their families after childbirth, when they are overwhelmed by the exhausting care for the newborn. The present study reveals how important it is for health care professionals to broaden their perception and care provided after childbirth for women with obesity so they may experience an improvement in their quality of health and of life.

## Resumo

**Objetivo** Explorar as vivências de mulheres com obesidade sobre o autocuidado e os cuidados recebidos da família e da equipe de saúde após o parto.

**Métodos** Estudo clínico-qualitativo realizado no Ambulatório de Revisão Puerperal do Hospital da Mulher da Universidade Estadual de Campinas, Brasil. A amostra foi selecionada de acordo com os critérios de saturação, com 16 mulheres com obesidade até 6 meses após o parto.

**Resultados** A análise de conteúdo compreendeu três categorias: 1) autocuidado pós-natal; 2) apoio familiar para a mulher após o parto; e 3) atenção do serviço de saúde à mulher com obesidade no pós-parto.

**Conclusão** As mulheres com obesidade necessitam de acolhimento e do apoio da equipe de saúde e de suas famílias após o parto, quando são absorvidas pelo cuidado exaustivo do recém-nascido. Este estudo revela o quão importante é para os profissionais de saúde ampliar sua percepção e cuidado após o parto às mulheres com obesidade para que estas possam melhorar sua qualidade de saúde e de vida.

## Introduction

The period after birth is a critical moment of transition and of physiological and psychological adaptations, which leaves women more susceptible to physical and emotional intercurrences.^1^ ^2^ The new maternity brings many challenges: besides her physiological recovery, the woman has to deal with the routine of caring for her baby and for herself. During this period, significant changes in her life demand new challenges, such as the acceptance of a new body image, sleep deprivation, adjustmentments in family relationships, as well as changes in her professional life, and in her health care and dietary care.^3^

A continuum of maternal and newborn care is essential to guarantee maternal and neonatal physical and mental health, irrespective of any complications at birth. This care requires a support network on which these women have relied throughout their lives: family, community and healthcare services.^4^

The postpartum period is considered by some authors^3^ as the 4th gestational trimester. It is a perspective that considers the woman and her baby as still being a mutually-dependent unit, linked both physiologically and behaviorwise. The intention behind insisting on the postpartum period as the “4th trimester” is to encourage actions to support women and their families during this critical period.^3^ ^5^

The innumerable physiological, psychological and social changes that take place in the lives of women after childbirth constitute a learning process of changes in lifestyle. The development of public health interventions for this specific population, at this point in their lives, when they are so busy, remains a challenge.^6^

Modern families are not receiving sufficient or quality support from family members or friends. Home visits by relatives and friends can help improve the mother's emotional wellbeing and self-esteem, as well as competency, family functioning, father-son/daughter relationship, and problem-solving.^7^

The mother's well-being after childbirth is greatly influenced by her psychosocial state, as well as by family support and by her environment.^8^ Having a baby brings emotional experiences to a woman's life. New psychological elaborations are needed, and some women feel more vulnerable to psychological problems during this period. Feelings of being overburdened and insecure about their ability to be a mother are linked to distress in the postnatal period.^8^

Self-care is an important component of motherhood.^9^ Time, limited resources and difficulty to accept help have been identified as obstacles to women's ability to care for themselves.^10^

The present study endeavors to explore the experiences of women with obesity vis-à-vis their self-care and the care they receive during the postnatal period, both from family members and the healthcare team. We define self-care here in its broadest meaning, as any care an individual takes towards him/herself. Thus, we try to identify aspects that can enable healthcare professionals to offer comprehensive care suited to women with obesity after childbirth.

## Methods

The clinical qualitative method was used,^11^ ^12^ which enables us to understand the emotional experiences of people involved in a healthcare setting. A fundamental part of this methodological structure is the interviewee's discourse. In this case, the scientific investigation is made based on the significance the interviewee attributes to the experiences, based on the premise that this is an efficient way of learning and inferring results that reveal the nexus of meanings.^12^ The clinical qualitative methodhas three particularities that define it: a) existentialist attitude: appreciation of the angst and anxiety arising from falling ill; b) clinical attitude: appreciation of the reception of the emotional suffering of a person and the desire to provide help; c) psychoanalytic attitude: appreciation of the elements underlying the interview, also admitting that unconscious elements are present in the interviewer-interviewee relationship.

### Setting

The present research was performed at the Postnatal Outpatient Clinic of Hospital da Mulher, Universidade Estadual de Campinas, a tertiary public teaching hospital, in Southeastern Brazil, which is a national benchmark in public care for women's and neonatal health. To this end, it relies on a multi-professional and interdisciplinary team, and it also promotes teaching, research and further education. The Postnatal Outpatient Clinic monitors postpartum women. The initial stage in the clinical qualitative research is acculturation,^12^ through which the researcher establishes a direct relationship with the population to be studied. The main researcher went to the Postnatal Outpatient Clinic for three months (between January and April 2016). The information gathered in this stage (the perceptions of the researcher and the reports of dialogues with the professionals or women after childbirth) were recorded in a field diary and used to formulate the questions initially proposed for the interviews.

### Participants

The selection of the sample was intentional: women over 18 years of age; up to 6 months after delivery; and with body mass index (BMI) ≥ 30 Kg/m^2^ before pregnancy were included. Women who were not breastfeeding were excluded. The sample was selected using the information saturation criteria,^13^ after discussion and validation with two research groups. The participants were women from the Postnatal Outpatient Clinic, and they were selected according to data recorded on the same day as their medical consultation. They were approached face to face by the interviewer (the main researcher), and were invited to take part in the study by means of an interview.

### Data Collection

The data was collected at the Outpatient Clinic, and the interviews took place between April and August 2017. All of the participants signed an informed consent form before the interviews, which were held in a private room, thus guaranteeing confidentiality. A single, semi-directed interview was performed with each participant, with open-ended questions allowing for depth,^14^ developed based on a script that was not rigid, thus enabling the interviewer to make the necessary adaptations based on the information provided by the interviewee. We have selected the questions pertaining to the theme of the present article that were made during the interview.

Trigger question: Tell me a little about how you have been feeling since your baby was born.

- Are you taking care of yourself?
- In what ways do you feel cared for?
- Do you have anyone to take care of you at home?
- How do you think the healthcare team can help you at this time?

### Data Analysis

Data analysis followed the seven steps described in the analysis of clinical qualitative content: 1) editing of the material: transcription of recorded interviews and convergence with material recorded in the field diary; 2) free-floating reading of the collected material: reading of material while suspending directed attention; 3) comments and impressions: taking notes and highlighting on the right hand margin of the transcript; 4) subcategorization and categorization: group and name significant speech within the same theme; however, the different categories contain heterogeneous ideas; 5) discussion with academic peers about the analyzed material; 6) category definition: refinement of the categories; and 7) validation of the analyzed material together with peers.

For the content analysis of the field research, the transcriptions of the interviews were performed by one of the co-authors. The editing of the written material, based on the transcriptions of the interviews and field analysis, was performed by the main researcher and author of the present study. At a later date, all of the material was read separately by the two independent researchers. Both completed the first stages of content analysis and comments individually. Following this, together they definedthe categories, which were also discussed with the research advisors and then presented and validated by two research groups.

The research was approved by the Ethics Committee of the Universidade Estadual de Campinas and the Brazilian National Board of Health in February 2017 (under the number CAAE62565116.3.0000.5404). The COREQ Checklist was also used for the present study.

## Results

The 16 women approached agreed to take part in the study. There were no refusals (Table 1).

**Table 1:** Characteristics of women with pre-pregnancy obesity after childbirth

Participants	Age (years)	Postpartum month	Weight (Kg)	Body mass index
P1	22	5	93	36.3
P2	34	2	118	45.5
P3	33	5	99	33.5
P4	23	2	94	31.8
P5	26	1	97	32.8
P6	23	4	105	37.6
P7	34	2	91	34.7
P8	29	3	94	32.9
P9	27	1	79	31
P10	29	4	96	31
P11	43	2	99	34.3
P12	39	3	84	35.4
P13	23	5	142	52.2
P14	29	2	79	31.2
P15	36	3	85	32.8
P16	20	2	82	30.5

The clinical qualitative content analysis revealed three categories: 1) postnatal self-care; 2) support from the family; and 3) postnatal healthcare services for women with obesity.

### Postnatal Self-care

The interviewees revealed a desire to take care of themselves, but the lonely routine with the baby made it difficult for them to think of doing anything for themselves. Caring for the newborn was a priority, which is perfectly natural at this stage in which the helplessness of the baby demands a huge, intense effort:

I don't take much care of myself. I have to admit I am not very vain, and now, less than ever, but I would like to […] the weight problem is something I would like to [deal with] because weight brings a lot of problems: it brought hypertension, it brought gestational diabetes, it brings, could bring me other problems that I don't want to deal with, I want to be healthy so she will be, you understand? *(Participant 11)*

Ah, I'm more worried about the baby […]. Not so much about myself, more about him. *(Participant 2)*

The breastfeeding routine and other care measures for the newborn can aggravate this situation and constitute an excuse for not thinking about or caring for themselves. We perceive that the issue of self-care, for some of these women, was not part of their routine long before pregnancy. The interviewees associated the word self-care with vanity rather than a health issue.

Ah, it's complicated, you see, I just let myself go: it's hair, nails, and now I don't even go out. I just stay at home with him [the baby]. And there's no way I can go to the beauty parlor; you have to have time to have your nails done, have your hair done, right? And up until now, I haven't managed that, his feeding time is on demand, right? *(Participant 4)*

From the health point of view, we perceived that despite identifying the postnatal period as a maturing process, they reveal a sense of negation of themselves in favor of the baby's emotional state, a feeling of their non-existence as ‘beings’ at this moment. They experience this process as something natural, because they feel that in some way they were already abnegating themselves in favor of pleasing others.

[…] We are trapped in a corner over there and you say, no, you are going to live for others and not for yourself. We have to understand ourselves and then the others, because if we are not well, we can't help others. Do you understand? Especially when we are mothers, there are times when we have to help, but if we are well, our child is well. During this 4-month phase [of the baby], they feel everything you have, normally I'm well, but the day when I was ill, the baby became ill, the day I'm feeling poorly, the baby feels a bit poorly. *(Participant 6)*

The interviewees reveal the importance of this stage after childbirth and how powerful they are to bring about changes, as long as they have the support of family members, friends and/or healthcare professionals. Encouraging the positive changes arising from pregnancy, so that these accomplishments continue after childbirth, is a way of guaranteeing the health of the woman. The subjective experiences, the sensation of maturing, and the intense care for the baby are important factors in insuring they are conscious of the need for self-care.

So I got it into my head, I myself am going to change, at the right moment, so I had planned the change so when he came, he helps me too and encourages me, because it is good to have an incentive in life, isn't it? *(Participant 9)*

Ah, how people talk, my God! How you are prettier, you lost weight, see! And I feel better, something like tiredness, that kind of thing, it's much better, [your] disposition *(Participant 14)*

### Support from the Family

The postpartum period marks a change in the women's attachment to healthcare services and family relationships. They reported a feeling of loss of the attention and care that they had received during pregnancy. This is experienced as a sudden and violent disruption, which corroborates the feeling of loneliness and helplessness.

Things that happen after pregnancy that make us feel so out of it that we think that nobody is helping us, but they are, you see? We don't feel cared for but they are taking care [of us]. *(Participant 6)*

Family members, in addition to helping with the routine at home and with the baby, can also provide support and care, while embracing the insecurities and helplessness felt by the woman during the postpartum period.

My father wanted to come and spend time with me, and I said “please come, dad”; then, my friend said “but your dad will not change diapers,” but I know that my father, he is caring […]. And that he will take care of me. *(Participant 4)*

Participants with strong family support reported the ability to organize meals in a more routine fashion, while those who did not have this support sought more practical and often unhealthy solutions.

I don't have much time to go to the supermarket, as fruit and vegetables have to be bought at least weekly […]. So, we are eating lots of tinned, fried, or preserved food; it's bad, but unfortunately… *(Participant 4)*

It is a great challenge for the woman after childbirth and those surrounding her to balance support and care for this woman, without depriving her of her autonomy. The family can be more aware and available to meet the physical and emotional needs of the woman: a caring gesture, the preparation of food, listening to her, holding her, always taking care not to see her as being fragile or less capable of making decisions about her life and that of her child(ren). As the woman finds herself in a period of greater emotional vulnerability, the care provided by the family can be seen as invasive or a threat to her autonomy.

Yes. I know that they are being excessively zealous towards me, I am grateful because few families, few pregnant women or new mothers get the opportunity of having their family close by, to take care of a newborn child; I'm lucky but it bothers me that I can't be in charge of my own life. *(Participant 11)*

The relationships with partners were approached many times during the interviews. The women spoke of the changes in their relationships and of how they identified that their partners' behavior in relation to childcare was very different from their own, which was reinforced culturally, and often by themselves, with feelings of guilt about delegating the care of their children to their partners.

It's difficult to find a father who helps a lot, who accepts the routine, who bathes and dresses the baby, wow! It is difficult to find, most do not want to know, but I think it is cultural. *(Participant 6)*

There is no more conversation […] we love each other and we get on well, but that's the way it is […] [my partner] cared for me and suddenly stopped, we miss it, it's the same as when something is taken away from you. *(Participant 3)*

### Postnatal Healthcare Service for Women with Obesity

The interviewees reported strong affective bonds with their antenatal team, with difficulties in disengaging themselves from their care. This caused an impact on the eating habits of these women, who reported that it would have been easier to lose weight and maintain a healthy eating pattern under the constant supervision of the team.

Here [at this healthcare service] I was losing weight without realizing it; it was my dream. I tried to keep this going, as my intention was to lose weight […] when you already have a tendency to enjoy eating, over the course of days I went back to my previous pattern. Eating a lot. *(Participant 11)*

During the postpartum period, follow-up at the healthcare service becomes less frequent and focused on contraception and breastfeeding. This was considered a negative experience, as they had to deal with the loss of this bond. They reported the need for a support network, which should include both family members and the healthcare service. The development of not only consultations, but also approaches that enable a discussion about subjectivity, self-care, food and weight are required.

I really needed dietary follow-up, an incentive with someone saying you are doing everything right […] because when you look at yourself, you see that you really have that willpower, you are putting faith in yourself that you will achieve it and improve your self-esteem. *(Participant 8)*

[…] The healthcare team needs to provide this help to the mother, as it really is too much for the mother. *(Participant 11)*

**Fig. 1:**
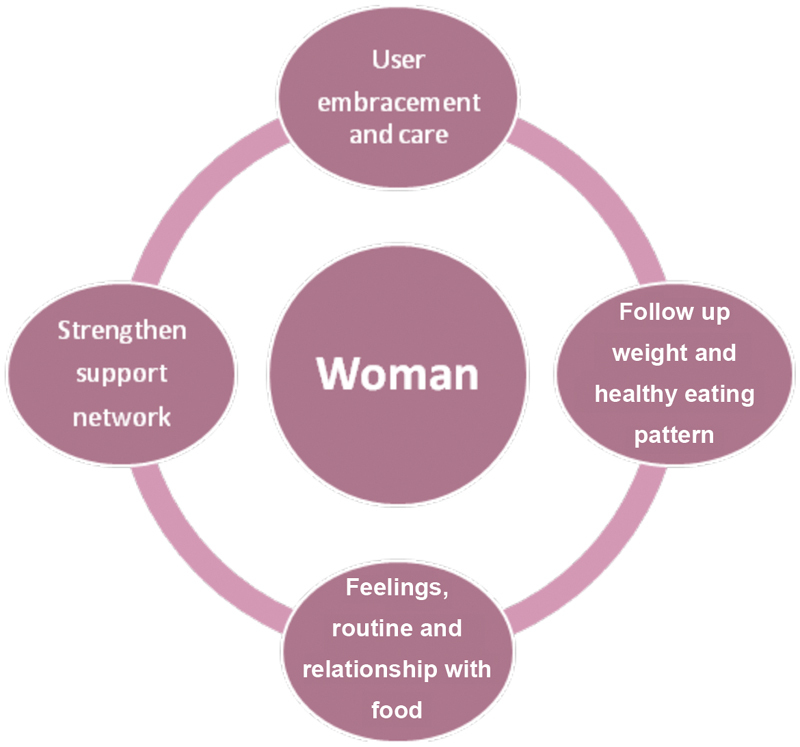
Postnatal care for women with obesity.

We perceived in the analysis of the interviews that women were keen to talk about their lives, showing interest in the possibility of being heard and welcomed. During the interviews, the moment in which the participants were most frequently emotional was when they were asked if they felt cared for. They revealed experiences of great solitude and a very strong desire that their relatives and health team perceive, understand and care for them without taking away their autonomy (Fig. 1).

## Discussion

Our results show that women with postnatal obesity tend to neglect care for themselves as caring for the newborn takes priority. Our interviewees report an experience associated with mourning for themselves, for their life up to that point, and how they lose themselves in a kind of ‘temporary depersonalization.’ These results are similar to those of other studies with postnatal women, irrespective of their BMI.^10^ ^15^ In their experience of motherhood, even women considered psychologically healthy experience a psychological withdrawal, giving up part of their interests as well as themselves to guarantee the baby's care. Mother and child become an autonomous unit that makes it possible for the mother to identify her baby's needs, something that is impossible to be identified by other people or in other circumstances.^16^

However, in this discussion we would like to highlight that women with obesity deserve greater attention in the postnatal period because, as shown by the interviewees, before becoming pregnant these women had a behavior pattern of prioritizing the needs of others over their own. This could reveal a difficulty to perceive themselves in a positive manner. A woman with obesity already feels that she is seen in a bad light, both by herself and by others. Our results are compatible with those in the literature, which show that obesity is correlated with low self-esteem and low self-control, social stigma, and shame.^17^ ^18^ As a consequence, obesity and excess weight do not affect only the health, but also the individual's sociability. Obesity has a stigma, a form of social discrimination that can cause many negative psychological effects in an individual.^11^ ^19^

Another aspect to be emphasized in our study of the experiences of women vis-à-vis their self-care is that they show that this is an opportune moment for interventions, since they feel that the experiences of motherhood bring an important maturing, and that, since pregnancy, they have become more inclined to acquire new habits. In the literature, we find that interventions by women's healthcare teams should be present during the pregnancy and continue throughout the puerperium to ensure that new habits be maintained.^15^ ^20^ ^21^ ^22^ Price et al^22^ (2012) state that new mothers were more disposed and interested in talking about behavior and goals for their children, but that months after the birth, especially when returning to work, women begin to focus again on themselves, and there is a window of opportunity to talk about their goals and behavior.

Our study also points to a psycho-educational need on the part of the interviewees, since they limit self-care to esthetic issues, and do not perceive it as being linked to health care. Promoting health is linked to strengthening the subjects' autonomy; self-care, appreciation of subjective experiences, as well as of the sociocultural contexts in which the individuals find themselves.^23^ ^24^ The advances in health care can guarantee an improvement in women's quality of life, and are one of the greatest challenges of this century. We perceive that this ignorance may be associated with psychological and cultural issues, and that health care professionals play an important role in this process of awareness as to the importance of self-care in health.^25^

Bearing in mind the experiences related by women with obesity after childbirth in relation to themselves, their families and the health care team, a discussion about the network of care for these women would be relevant to develop strategies to provide support and care for them. The postpartum period is a critical moment, and it demands a continuum of maternal care in its different human dimensions.^26^

The intense care regarding the physical and emotional needs of the baby and his/her primitive psychological states also awakens/evokes states of primitive anxiety and a sense of internal solitude in the mother, as well as the mourning process a woman must face vis-à-vis her pregnancy and life prior to maternity. This concept of solitude was described by Klein^27^ (1984) as a feeling of loneliness irrespective of the external context, irrespective of being among beloved people and surrounded by love and attention.

Family relationships should be encouraged and strengthened at this time. It is important that families are aware of what constitutes this moment in a woman's life and her needs. The interviewees reported that these relationships can be conflictual and disrespectful, and cause further emotional overload to the mother. The literature describes how much a good family relationship can help these women have a better quality of life in the postnatal period. Price et al^22^ (2012) state that, during this period, women find many barriers to eating healthy, and when there is the constant presence of a family member, they are able to eat better and lead a healthier life.

It is very important that health care teams be aware of the family relationships of these women to identify failures in the family support network as well as when the care offered takes away their autonomy.

In order for women with obesity to become aware of the need for self-care, it is important for the team to begin with assertive comments and offer alternatives as to how this woman can care for herself, by showing that this is both an external and internal task. Our results correspond to those presented by Chugh et al^28^ (2013) in that the disposition to lose weight depends as much on self-motivation as on the incentive of the health professional. The study^28^ showed that the perception of the health professionals regarding the lowest level of motivation for these women to lose weight can make this process even more difficult.

The content of the care offered after childbirth must be developed in such a way as to include more priorities in women's health studies.^29^ ^30^ New mothers have multiple unmet needs, and health institutions should be aware of these needs and provide them with support by means of clear and precise information, so they do not feel alone, sheltering them in their search for information and rights.^3^ Moreover, women have different ideas, experiences and expectations about losing weight in the postnatal period. Health care professionals can take care of the needs of each woman to promote their autonomy and better results in their health and lifestyle.^31^

We have proposed a plan directed at healthcare professionals who care for women with postnatal obesity (Fig. 1). Our study also sought to provide tools for healthcare professionals during the follow-up of these women. The follow-up should make the women feel a sense of belonging and care; in it, weight and nutrition should be monitored, and the support network, whether from family, friends, or other women going through the postpartum period, must be strengthened, and environments in which these women are encouraged to talk about their feelings, routine and relationship with food should be created. The idea is that the health service provides centrality for the women and their experiences. The women showed throughout the interviews that more important than talking was the feeling that they were being heard.

Fig. 1 proposes a model of care for the psychological aspects of women with obesity. We emphasize the care already included in the gynecological and obstetric teams' routines of breastfeeding, contraception and clinical care.

## Conclusion

The postnatal period is a landmark in the physiology, the social life and psychological state of women, which is capable of transforming their subjectivity and identity. This period requires interventions by health professionals. The women with obesity already feel discriminated against, both by themselves and by others, which also brings risks of psychopathological disorders and risks regarding their physical health and weight, which are more evident after childbirth. The women with obesity need attention as well as the presence of the health team and of family members in order to take better care of their health, at a moment when women are already overwhelmed by the exhausting care for the newborn. Effective strategies are needed for women with obesity in the postnatal period, thus guaranteeing their quality of life.
